# Gp130-Mediated STAT3 Activation Contributes to the Aggressiveness of Pancreatic Cancer through *H19* Long Non-Coding RNA Expression

**DOI:** 10.3390/cancers14092055

**Published:** 2022-04-19

**Authors:** Norihiko Sasaki, Kazumi Hirano, Yuuki Shichi, Fujiya Gomi, Hisashi Yoshimura, Akira Matsushita, Masashi Toyoda, Toshiyuki Ishiwata

**Affiliations:** 1 Research Team for Geriatric Medicine (Vascular Medicine), Tokyo Metropolitan Institute of Gerontology, Tokyo 173-0015, Japan; sasanori@tmig.or.jp (N.S.); mtoyoda@tmig.or.jp (M.T.); 2 Molecular Neurophysiology Research Group, Biomedical Research Institute, The National Institute of Advanced Industrial Science and Technology (AIST), Ibaraki 305-8568, Japan; kazumi-hirano@aist.go.jp; 3 Division of Aging and Carcinogenesis, Research Team for Geriatric Pathology, Tokyo Metropolitan Institute of Gerontology, Tokyo 173-0015, Japan; y_shichi@tmig.or.jp (Y.S.); gomif@tmig.or.jp (F.G.); 4 Laboratory of Physiological Pathology, Department of Applied Science, School of Veterinary Nursing and Technology, Nippon Veterinary and Life Science University, Tokyo 180-8602, Japan; yoshimura-h@nvlu.ac.jp; 5 Department of Gastrointestinal and Hepato-Biliary-Pancreatic Surgery, Nippon Medical School, Tokyo 113-8603, Japan; akira-matsushita@nms.ac.jp

**Keywords:** gp130, STAT3, pancreatic cancer, cancer stem cell, invasion, EMT, *H19*

## Abstract

**Simple Summary:**

The signal transducer and activator of transcription 3 (STAT3) activation correlate with the aggressiveness of pancreatic ductal adenocarcinoma (PDAC). We demonstrated that the autocrine/paracrine interleukin-6 (IL-6) or leukemia inhibitory factor (LIF)/glycoprotein 130 (gp130)/STAT3 pathway contributes to the maintenance of stemness features and membrane-type 1 matrix metalloproteinase (MT1-MMP) expression, and modulates transforming growth factor (TGF)-β1/Smad signaling-mediated epithelial-mesenchymal transition (EMT) and invasion through regulation of TGFβ-RII expression in PDAC cancer stem cell (CSC)-like cells. Furthermore, we demonstrated that p-STAT3 acts through the IL-6 or LIF/gp130/STAT3 pathway to access the active promoter region of metastasis-related long non-coding RNA *H19* and contribute to its transcription in CSC-like cells. Therefore, the autocrine/paracrine IL-6 or LIF/gp130/STAT3 pathway in PDAC CSC-like cells exhibiting *H19* expression is considered to be involved in the aggressiveness of PDAC, and inhibition of the gp130/STAT3 pathway is a promising strategy to target CSCs for the elimination of PDAC (146/150).

**Abstract:**

Signaling pathways involving signal transducer and activator of transcription 3 (STAT3) play key roles in the aggressiveness of pancreatic ductal adenocarcinoma (PDAC), including their tumorigenesis, invasion, and metastasis. Cancer stem cells (CSCs) have been correlated with PDAC aggressiveness, and activation of STAT3 is involved in the regulation of CSC properties. Here, we investigated the involvement of interleukin-6 (IL-6) or the leukemia inhibitory factor (LIF)/glycoprotein 130 (gp130)/STAT3 pathway and their role in pancreatic CSCs. In PDAC CSC-like cells formed by culturing on a low attachment plate, autocrine/paracrine IL-6 or LIF contributes to gp130/STAT3 pathway activation. Using a gp130 inhibitor, we determined that the gp130/STAT3 pathway contributes to the maintenance of stemness features, the expression of membrane-type 1 matrix metalloproteinase (MT1-MMP), and the invasion of PDAC CSC-like cells. The gp130/STAT3 pathway also modulates the transforming growth factor (TGF)-β1/Smad pathway required for epithelial-mesenchymal transition induction through regulation of TGFβ-RII expression in PDAC CSC-like cells. Furthermore, chromatin immunoprecipitation assays revealed that p-STAT3 can access the active promoter region of *H19* to influence this metastasis-related long non-coding RNA and contribute to its transcription in PDAC CSC-like cells. Therefore, the autocrine/paracrine IL-6 or LIF/gp130/STAT3 pathway in PDAC CSC-like cells may eventually facilitate invasion and metastasis, two hallmarks of malignancy. We propose that inhibition of the gp130/STAT3 pathway provides a promising strategy for targeting CSCs for the treatment of PDAC.

## 1. Introduction

Pancreatic ductal adenocarcinoma (PDAC) is one of the most lethal malignancies, with a 5-year survival rate of approximately 11%, and PDAC is the 3rd leading cause of cancer-related deaths in the USA and the 4th leading in Japan [1]. Surgery is the only treatment option; however, 80% of PDAC patients are at an advanced stage of cancer at diagnosis [2]. Even in patients who undergo surgery, local recurrence of cancer and development of distant metastases frequently occur [3]. In the USA, PDAC is expected to be the second leading cause of cancer-related deaths by 2030 [4]. Recent studies have shown that heterogeneous cancer cells, including a small number of cancer stem cells (CSCs), form cancerous tumors [5,6,7]. CSCs contribute to tumor initiation, growth, and metastasis and are resistant to chemotherapy or radiotherapy. Based on the concept that CSCs are responsible for tumor self-renewal, the elimination of CSCs could lead to the disappearance of cancer cells [8].

Signal transducer and activator of transcription 3 (STAT3) signaling pathways have been suggested to play a key role in tumorigenesis, progression, and drug resistance in several human malignancies, including leukemia, lymphomas, hepatocellular carcinoma, and esophageal, lung, prostate, bladder, and breast cancers [9,10]. STAT3 is an important regulator of stem cell self-renewal and cancer cell survival in the animal model of PDAC [11,12]. In human breast tumors, phosphorylation of STAT3 enhances the transcription of downstream target genes that promote angiogenesis, invasion, and epithelial-mesenchymal transition (EMT) [13]. Targeted inhibition of STAT3 is considered to be an effective therapeutic strategy for PDAC. A recent study has shown that a new small-molecule inhibitor of STAT3 suppresses tumor growth and metastasis and significantly prolongs the survival of tumor-bearing mice in animal models of PDAC [14]. STAT3 signaling is activated by the binding of various IL-6 family cytokines, such as interleukin-6 (IL-6) or leukemia inhibitory factor (LIF), to their cell surface receptors, including glycoprotein 130 (gp130) (a common subunit of the receptor complexes), thus leading to the phosphorylation of STAT3 [15]. While individual IL-6 family members also regulate AKT and ERK signaling through gp130, besides STAT3, the relative strength of activation of specific pathways can differ depending on the cytokine, cell type, and physiological context [16,17,18]. For example, oncostatin M (OSM), but not IL-6 or LIF, induces the tyrosine phosphorylation of the Shc isoforms p52 and p66 and their association with Grb2, resulting in a stronger activation of ERK signaling in hepatoma cells [19]. The LIF/gp130/STAT3 signaling pathway is well recognized to contribute to the maintenance of stemness in mouse embryonic stem cells [20,21]. In CSCs, the IL-6/gp130/STAT3 signaling pathway has been demonstrated to be involved in the regulation of CSC properties such as self-renewal, stemness marker expression, and tumor cell growth in prostate cancer [22], glioma [23], endometrial cancer [24], and breast cancer [25]. Blocking the gp130/STAT3 signaling pathway may eliminate CSCs and help prevent cancer, due to the important role that this pathway plays in maintaining CSC properties such as self-renewal abilities in carcinogenesis.

Long non-coding RNAs (lncRNAs) are mRNA-like transcripts that contain more than 200 ribonucleotides [26]. LncRNAs regulate gene expression levels by participating in epigenetic modification and control of transcription, RNA processing, translation, and post-translational modification [26]. LncRNAs play important roles in carcinogenesis, proliferation, and metastasis in various types of cancers [27,28,29,30]. The human *H19* gene is located on chromosome 11p15.5 and encodes an imprinted lncRNA [31]. Recent studies have shown that *H19* enhances invasion and metastasis in several malignancies, including bladder cancer [32,33], glioma [34], osteosarcoma [35], acute myeloid leukemia [36], breast cancer [37,38], non-small cell lung cancer [39], gastric cancer [40], and pancreatic cancer [41] while suppressing the aggressiveness of hepatocellular carcinoma [42] and prostate cancer [43]. We have recently reported that *H19* was the most highly expressed (82.4-fold) ncRNA in a lung metastasis-derived human PDAC cell line, compared to the parental PDAC cell line PANC-1, and that *H19* inhibition decreased lung and liver metastases in immunodeficient NOG mice [44]; thus, *H19* is expected to be a novel therapeutic target for pancreatic cancer metastasis. Therefore, it is important to clarify the mechanisms underlying *H19* regulation. To date, the signaling pathways and/or transcription factors that directly control the expression of *H19* have not been clarified. We previously reported that *H19* is increased in PDAC sphere cells in which CSCs are enriched [45]. Therefore, we speculated that stemness-related signaling might be involved in the expression of *H19*.

Previous reports indicate that CSCs are enriched in these spheres and play an important role in the biological aggressiveness of PDAC [46,47,48]. Sphere cells formed under 3D-culture conditions have been characterized as CSC-like cells and have been used for CSC research. Previously, we demonstrated that serum-containing media yielded the most rapid formation of large spheres (including CSCs) in PDAC cells [49,50]. To date, it has not been clarified whether IL-6 or LIF/gp130/STAT3 signaling pathways contribute to the maintenance of CSCs in PDAC. Furthermore, there have been no reports regarding stemness-related signaling of *H19*. In the present study, we investigated the correlation between the involvement of IL-6 or LIF/gp130/STAT3 signaling in PDAC CSC-like cells and *H19* expression.

## 2. Materials and Methods

### 2.1. Cell Culture

The human PDAC cell lines PK-1, PANC-1, and PK-59 were obtained from the Cell Resource Center for Biomedical Research, Institute of Development, Aging, and Cancer, Tohoku University (Sendai, Japan). The PK-8 human PDAC cell line was provided by RIKEN BRC through the National Bio-Resource Project of MEXT, Japan. Cells were grown in a growth medium (RPMI 1640 medium containing 10% fetal bovine serum) at 37 °C under a humidified 5% CO_2_ atmosphere. For sphere formation by 3D culture, cells in the growth medium were plated at 1.0 × 10^4^ cells/well or 3.0 × 10^3^ cells/well into 24-well ultra-low attachment plates (Corning Inc., Kennebunk, ME, USA) or 96-well ultra-low attachment plates (Thermo Fisher Scientific, Waltham, MA, USA). The spheres were aspirated after seven days using micropipettes and placed in microcentrifuge tubes for use in further experiments. To inhibit gp130 ligand-triggered signaling, the cells were treated with SC144 (Selleck Chemicals, Houston, TX, USA) or DMSO as a vehicle control. The size (area) of spheres was quantified using ImageJ software (National Institutes of Health, Bethesda, MD, USA).

### 2.2. Transfection of Short Interfering RNA (siRNA) Targeting IL-6 or LIF

The cells were plated at a density of 2 × 10^5^ cells into 35-mm dishes and transfected 24 h later with 5 nM siRNAs targeting IL-6 (5-GAA CGA AUU GAC AAA CAA ATT-3), LIF (5-UGC CUC UAC UUG UCC CUU UTT-3), or Silencer negative control siRNA (siCont) using Lipofectamine™ RNAiMAX Transfection Reagent (Thermo Fisher Scientific) according to the manufacturer’s protocol.

### 2.3. Generation of Stable H19-Overexpressing Cells

The *H19* full-length cDNA (2.3 kb) cloned into pcDNA3.1(−) vector (a generous gift from Dr. Eric Adriaenssens, Science and Technology, Lille 1 University, France) and empty vector were transfected into PANC-1 cells using FuGENE HD transfection reagent (Roche Diagnostics, Mannheim, Germany) according to the manufacturer’s instructions. Briefly, 2 × 10^5^ cells/mL were transfected with 2 μg of DNA using FuGENE HD, and the cells were passaged and cultured with 1 mg/mL of geneticin (Sigma-Aldrich Corporation, St. Louis, MO, USA) [44,45].

### 2.4. Fluorescence-Activated Cell Sorting (FACS) Analysis

Cells were harvested, and dissociated single cells were incubated on ice for 30 min with PE-conjugated anti-human IL-6 receptor α (IL-6Rα) antibody (BioLegend, San Diego, CA), PE-conjugated anti-human LIF receptor (LIFR) antibody (Becton Dickinson, Franklin Lakes, NJ, USA), PE-conjugated anti-human gp130 antibody (BioLegend), or PE-conjugated isotype control (Becton Dickinson) diluted in FACS buffer (0.5% (*w*/*v*) BSA and 0.1% (*w*/*v*) sodium azide in PBS). After washing, the cells were analyzed using a FACSAria™ cell sorter (Becton Dickinson). The mean fluorescence intensity (MFI) was calculated by subtracting the intensity of the control.

### 2.5. Real-Time Quantitative Polymerase Chain Reaction (qPCR)

Total RNA was isolated from the cells using the RNeasy Plus Mini Kit (QIAGEN, Hilden, Germany) and subsequently reverse-transcribed using the ReverTra Ace^®^ qPCR RT Kit (Toyobo, Osaka, Japan) according to the manufacturer’s instructions. Real-time qPCR was performed using the Power SYBR ^®^ Green kit (Applied Biosystems, Foster City, CA, USA) and StepOnePlus™ real-time PCR system (Applied Biosystems, Foster City, CA, USA). *β*-*Actin* was used as an internal control. The primer sets used for qPCR are listed in Table S1. Real-time qPCR analysis for *H19* was performed using TaqMan Fast Universal PCR Master Mix (Life Technologies Corporation, Carlsbad, CA, USA) and TaqMan Gene Expression Assays (Life Technologies Corporation), with 18S rRNA serving as an internal control.

### 2.6. Sphered Cell Proliferation Assay

Cells were cultured in a growth medium at a density of 3 × 10^3^ cells/well in 96-well ultra-low attachment plates and incubated for 7 days. Adenosine triphosphate (ATP) assays were used to examine proliferation using the CellTiter-Glo^®^ 2.0 assay (Promega, Madison, WI, USA) according to the manufacturer’s protocol.

### 2.7. Immunoblotting

Cells were lysed with lysis buffer (50 mM Tris-HCl pH 7.4, 150 mM NaCl, 1.5 mM MgCl_2_, 5 mM EDTA, and 1% Triton™ X-100) containing protease and phosphatase inhibitor cocktails. The samples prepared as described above were separated by SDS-PAGE and then transferred onto PVDF membranes (Merck Millipore, Billerica, MA, USA). After blocking, the membranes were incubated with the primary antibodies. All antibodies used in this study are listed in Table S2. Membranes were then incubated with the appropriate peroxidase-conjugated secondary antibodies (Cell Signaling Technology, Danvers, MA, USA), washed, and developed using ECL Prime reagents (GE Healthcare, Piscataway, NJ, USA). Image densitometry was performed using the ImageJ software (National Institutes of Health, Bethesda, MD, USA). Full-blot images with molecular weight markers are shown in Figure S7.

### 2.8. Anti-Drug Resistance Assay

Cells (3.0 × 10^3^ cells/well) were plated in 96-well ultra-low attachment plates with a growth medium with or without SC144. Each anticancer drug was administered at the indicated concentration after 7 days of culture, and cell growth rates were measured by ATP assays 4 days after treatment with the anticancer drug. Cell viability was calculated as the percentage of luminescence in drug-treated cells relative to that in untreated control cells.

### 2.9. Invasion Assay

Invasion assays were performed using Corning Matrigel invasion chambers (pore size: 8 μm; Discovery Labware Inc., Bedford, MA, USA). Cells were plated at a density of 1 × 10^5^ cells/500 μL onto the upper surface of the inserts, and 24 h later, cells that migrated through the membrane to the lower surface of the filter were fixed and stained with a Diff-Quick staining kit (Polysciences, Inc., Warrington, PA, USA) and then counted under a light microscope.

### 2.10. 3D Invasion Assay

Cells (3.0 × 10^3^ cells/well) in the growth medium were plated into 96-well ultra-low attachment plates. After incubation for 7 days, the growth medium was gently removed from the spheroid-containing plates and 100 μL of Matrigel solution in PBS was gently dispensed into the U-bottom wells. The plates were incubated at 37 °C to allow the Matrigel solution to solidify, and 100 μL of growth medium was then gently added to each well. The spheres were photographed using a phase-contrast microscope (Nikon, Tokyo, Japan) at an appropriate incubation time. We then calculated the area of the sphere at each experimental time point compared to the areas at 0 h to evaluate the invasion ability.

### 2.11. Chromatin Immunoprecipitation (ChIP) Analysis

ChIP was performed according to the protocol published by Agilent Technologies (Santa Clara, CA, USA) with some modifications. Briefly, the cells were fixed with an 11% formaldehyde solution for 10 min. After harvesting the fixed cells, they were lysed in lysis buffer and sonicated using a Misonix XL2020 sonicator (MISONIX Inc., Farmingdale, NY, USA, http://misonix.com, accessed on 18 January 2022) until the DNA fragments were 200–600 bp in length. Aliquots of 3.0% of the total volume were stored as input at −20 °C until use. Immunoprecipitation was performed at 4 °C overnight with anti-trimethylation of histone H3 Lys 4 (H3K4me3) (ab8580; Abcam, Cambridge, UK), histone H3 acetyl Lys27 (H3K27Ac) (ab4729; Abcam), and p-STAT3 (Tyr705) (#9131; Cell Signaling Technology). DNA/beads were washed with a low salt buffer (20 mM Tris-HCl pH 7.4, 150 mM NaCl, 2 mM EDTA, 0.1% sodium dodecyl sulfate [SDS], and 1% Triton X-100) once and then further washed with a high-salt buffer (20 mM Tris-HCl pH 7.4, 400 mM NaCl, 2 mM EDTA, 0.1% SDS, and 1% Triton X-100) once before washing with RIPA buffer (50 mM HEPES-KOH, pH 7.6, 500 mM LiCl, 1 mM EDTA, 1% NP-40, and 0.7% Na-deoxycholate). Immune complexes were disrupted with direct elution buffer (50 mM Tris-HCl pH8.0, 10 mM EDTA, and 1% SDS), and the covalent links between immunoprecipitates and input chromatin were disrupted by incubation at 65 °C overnight. The DNA was further incubated with RNase A and proteinase K (Nacalai Tesque, Kyoto, Japan), purified by phenol extraction, and precipitated with ethanol. The DNA pellets were dissolved in Tris-EDTA buffer (10 mM Tris-HCl and 1 mM EDTA pH8.0). Co-immunoprecipitated DNA was detected by quantitative PCR using primers specific to the *H19* promoter region (Table S3). Two independent experiments were conducted.

### 2.12. Gene Correlation Analysis

The online database Gene Expression Profiling Interactive Analysis (GEPIA) (http://gepia.cancer-pku.cn/index.html, accessed on 18 January 2022) was used to analyze the correlation of the expression of gp130/STAT3 pathway-related factors with overall survival and *H19* expression correlated with gp130/STAT3 pathway-related factors in patients with PDAC. Gene expression correlation analysis was performed for the given sets of TCGA expression data by using GEPIA. The Spearman method was used to determine the correlation coefficients. *H19* was used as the x-axis, and other genes of interest are represented on the y-axis.

### 2.13. Statistical Analysis

Results are presented as mean ± SD, and the data between the two groups were compared using Student’s *t*-test or Welch’s *t*-test. One-way ANOVAs were performed to compare multiple pairing groups. Statistical analyses were performed using Microsoft Excel for Mac statistical analysis, version 3.0. Statistical significance was set at *p* < 0.05.

## 3. Results

### 3.1. Active gp130/STAT3 Pathway in PDAC Sphere Cells

**Figure 1:**
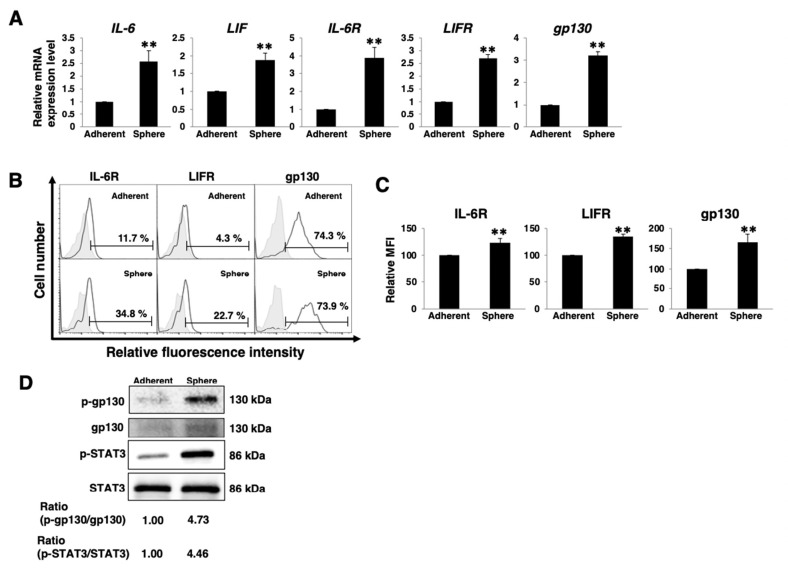
Active gp130/STAT3 pathway in PANC-1 sphere cells. (**A**) Real-time qPCR analysis of *IL-6*, *LIF*, *IL-6R*, *LIFR*, and *gp130* in PANC-1 cells cultured in 2D (adherent) or 3D (sphere) conditions. The results presented are normalized to values obtained for adherent cells (value = 1). Results are presented as means ± SD from three independent experiments. (**B**) FACS analysis of IL-6R, LIFR, and gp130 expression in PANC-1 cells cultured in 2D (adherent) or 3D (sphere) conditions. Controls are indicated by thin lines with gray color. (**C**) Cell surface levels of IL-6R, LIFR, and gp130 expression in PANC-1 cells cultured in 2D (adherent) or 3D (sphere) conditions. Mean fluorescence intensities (MFIs) relative to those of adherent cells are presented. Results are presented as means ± SD from three independent experiments. (**D**) Western blot analysis of p-gp130, gp130, p-STAT3, and STAT3 was performed in PANC-1 cells cultured in 2D (adherent) or 3D (sphere) conditions. Relative band intensity is provided. ** *p* < 0.01.

First, we compared the expression of gp130/STAT3 pathway-related factors, such as IL-6, LIF, IL-6R, LIFR, and gp130, between 2D (adherent) and 3D (sphere) cultured PDAC cell lines. Real-time qPCR analysis demonstrated that all examined factors were significantly upregulated in PANC-1 sphere cells (Figure 1A) and that almost all factors were upregulated in PK-1 sphere cells (Figure S1A). Cell surface expression of IL-6, LIFR, and gp130, as assessed by FACS analysis, revealed that all of these factors were expressed at higher levels in sphere cells of PANC-1 and PK-1 cells (Figure 1B,C and Figure S1B). Next, we examined the activation of the gp130/STAT3 pathway by immunoblotting. As presented in Figure 1D and Figure S1C, the phosphorylation of gp130 and STAT3 was increased in the sphere cells of PANC-1 and PK-1 cells. These results indicated that the gp130/STAT3 pathway is functional in certain PDAC cell lines, including PANC-1 and PK-1 cells. Therefore, most of the subsequent experiments with PDAC cell lines were performed using PANC-1 and PK-1 cells.

### 3.2. Dose-Dependent Growth Inhibition via gp130 Inhibitor in PDAC Sphere Cells

**Figure 2:**
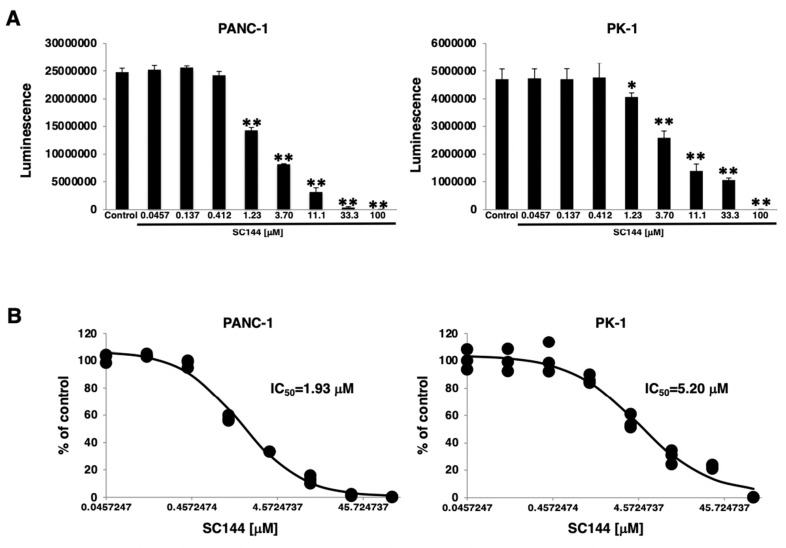
Dose-dependent growth inhibition via gp130 inhibitor treatment in PDAC sphere cells. (**A**) PANC-1 and PK-1 cells were treated with SC144 at the indicated concentrations in triplicate for 7 days in 3D-culture conditions, and the ATP assay was performed to analyze the growth of sphere cells (* *p* < 0.05, ** *p* < 0.01 vs. control). (**B**) IC_50_ values were measured for each cell line following 7 days under 3D-culture conditions with various concentrations of SC144.

The effect of the gp130 inhibitor, SC144, on PDAC sphere cell proliferation was investigated in PANC-1 and PK-1 cells. To investigate sphere cell proliferation, PDAC cells were cultured under 3D-culture conditions with varying concentrations of SC144 for 7 days. In both PANC-1 and PK-1 cells, cell viability was decreased significantly at an SC144 concentration of 1.23 μM (Figure 2A). The 50% inhibitory concentration (IC_50_) of SC144 was calculated from the cell survival curves of both cells. The IC_50_ values for the two cell lines were 1.93 μM (PANC-1) and 5.20 μM (PK-1) (Figure 2B).

### 3.3. Active gp130/STAT3 Pathway Is Involved in Stemness and Invasion of PDAC Sphere Cells

**Figure 3:**
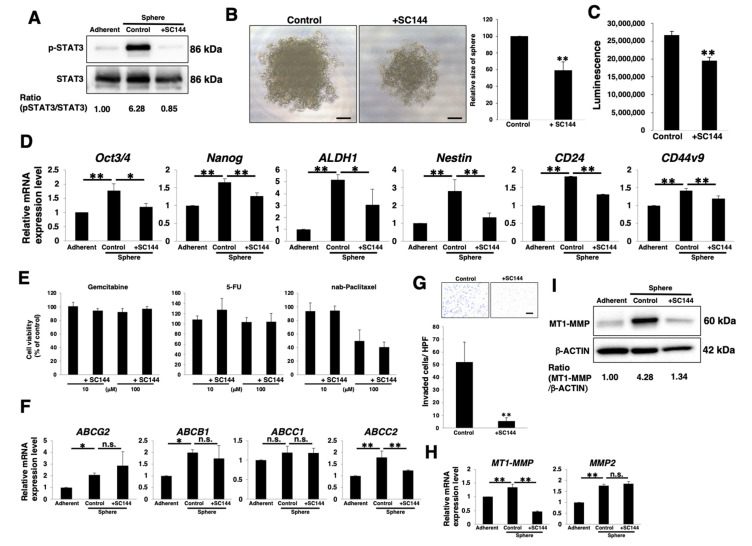
Active gp130/STAT3 pathway involved in stemness and invasion of PANC-1 sphere cells. PANC-1 cells were cultured for 7 days in 2D (adherent) or 3D (sphere) conditions with or without 1 μM SC144. The cells were harvested for use in the described experiments. In (**E**), as described in Materials and Methods, each anticancer drug was added to 7-day-cultured sphere cells, and the cells were further cultured for 4 days. (**A**) Western blot analysis of p-STAT3 and STAT3. Relative band intensity is presented. (**B**) Phase-contrast image of PANC-1 sphere cells in 96-well ultra-low attachment plates at day 7. The histograms show the relative sizes of spheres (control = 100). Results are presented as means ± SD from four sphere images. Scale bar: 5 μm. (**C**) ATP assay for growth rate of PANC-1 sphere cells. (**D**) Real-time qPCR analysis of stemness markers. Results are normalized to values obtained for adherent cells (value = 1). Results are presented as means ± SD from three independent experiments. (**E**) Anticancer drugs (gemcitabine, 5-FU, or nab-paclitaxel) resistance assay in PANC-1 sphere cells. The dose-response (10 or 100 μM) of PANC-1 sphere cells to each drug was determined using the ATP assay. (**F**) Real-time qPCR analysis of transporters. Results are normalized to values obtained for adherent cells (value = 1). Results are presented as means ± SD from three independent experiments. (**G**) Matrigel invasion assays were performed in PANC-1 sphere cells. Representative results from measurements of 12 fields are presented. Scale bar: 100 μm. (**H**) Real-time qPCR analysis of *MT1-MMP* and *MMP2*. Results are normalized to values obtained for adherent cells (value = 1). Results are presented as means ± SD from three independent experiments. (**I**) Western blot analysis of MT1-MMP and β-ACTIN. Relative band intensity is presented. * *p* < 0.05; ** *p* < 0.01, n.s.: not significant.

To clarify the correlation between the gp130/STAT3 pathway and stemness in PDAC sphere cells, we examined stemness features including sphere-forming ability, stemness marker expression, anticancer drug resistance, and invasion ability. Immunoblotting revealed that SC144 treatment inhibited the phosphorylation of STAT3 in PANC-1 sphere cells (Figure 3A), indicating that the gp130/STAT3 pathway is attenuated by SC144 treatment. Sphere formation was attenuated in SC144-treated PANC-1 (Figure 3B,C) and PK-1 sphere cells (Figure S2A). Real-time qPCR analysis demonstrated that all examined stemness markers were expressed at lower levels in SC144-treated PANC-1 sphere cells than in control sphere cells (Figure 3D). In contrast, two of the six markers (*Nanog* and *Nestin*) were expressed at lower levels in SC144-treated PK-1 cells (Figure S2B). These results suggest that the gp130/STAT3 pathway is involved in the expression of stemness markers in PDAC cells. To assess anticancer drug resistance in sphere cells, we used three anti-pancreatic cancer drugs, gemcitabine, 5-FU, and nab-paclitaxel. Only nab-paclitaxel was cytotoxically effective in PANC-1 sphere cells, and cell survival rates following the addition of nab-paclitaxel (100 μM) were approximately 50% (Figure 3E), an observation that was consistent with our previous report [51]; however, the survival rates were not significantly different in SC144-treated PANC-1 sphere cells following incubation with the above anticancer drugs at either 10 or 100 μM (Figure 3E). Furthermore, we examined the expression levels of four potential anticancer drug transporters. Real-time qPCR analysis revealed that the expression of *ABCG2*, *ABCB1*, and *ABCC1* was not significantly different between the control and SC144-treated PANC-1 sphere cells (Figure 3F). In contrast, *ABCC2* expression was reduced in SC144-treated PANC-1 cells (Figure 3F); thus, the gp130/STAT3 pathway is involved in regulating the expression of ABCC2 among the four anticancer drug transporters, but is not involved in the resistance to the anticancer drugs in PANC-1 sphere cells. Next, we examined the invasion ability of the cells. Invasion assays revealed that the number of invading cells was significantly reduced in SC144-treated PANC-1 and PK-1 sphere cells compared to that in control cells (Figure 3G and Figure S2C, respectively), indicating that the gp130/STAT3 pathway promotes cellular invasion. A key feature of invasion is increased production of matrix metalloproteinases (MMPs) [52,53]; MMP2 and MT1-MMP are known to be expressed in PDAC cells [54]. Therefore, we hypothesized that the gp130/STAT3 pathway promotes MMP expression and activity. Real-time qPCR revealed that *MT1-MMP* mRNA levels were significantly upregulated in PANC-1 and PK-1 sphere cells compared to those in adherent cells and were reduced in SC144-treated sphere cells (Figure 3H and Figure S2D, respectively). Furthermore, western blot analysis demonstrated that the protein levels of MT1-MMP were significantly reduced in SC144-treated PANC-1 cells (Figure 3I).

### 3.4. Autocrine/Paracrine IL-6 or LIF/gp130/STAT3 Pathway Involved in Stemness and Invasion of PDAC Sphere Cells

**Figure 4:**
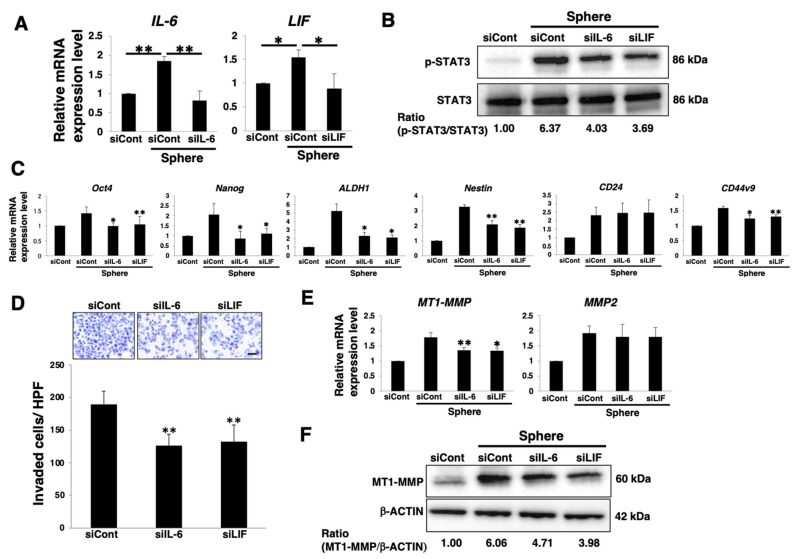
Autocrine/paracrine IL-6 or LIF/gp130/STAT3 pathways involved in stemness and invasion of PDAC sphere cells. PANC-1 cells were cultured for 7 days in 2D (adherent) or 3D (sphere) conditions after siRNA transfection. The cells were harvested and then used in the following experiments. (**A**) Real-time qPCR analysis of *IL-6* or *LIF*. Results are normalized to values obtained for adherent cells (value = 1). Results are presented as means ± SD from three independent experiments. (**B**) Western blot analysis of p-STAT3 and STAT3. Relative band intensity is presented. (**C**) Real-time qPCR analysis of stemness markers. Results are normalized to values obtained for adherent cells (value = 1). Results are presented as means ± SD from three independent experiments (* *p* < 0.05, ** *p* < 0.01 vs. siCont transfected sphere cells). (**D**) Matrigel invasion assays were performed in PANC-1 sphere cells. Representative results from measurements of 12 fields are presented (* *p* < 0.05, ** *p* < 0.01 vs. siCont transfected sphere cells). Scale bar: 100 μm. (**E**) Real-time qPCR analysis of *MT1-MMP* and *MMP2*. Results are normalized to values obtained for adherent cells (value = 1). Results are presented as means ± SD from three independent experiments (* *p* < 0.05, ** *p* < 0.01 vs. siCont transfected sphere cells). (**F**) Western blot analysis of MT1-MMP and β-ACTIN. Relative band intensity is presented.

Next, we examined whether the gp130/STAT3 pathway in PDAC sphere cells is activated by the autocrine/paracrine action of IL-6 or LIF. Knockdown of *IL-6* or *LIF* by siRNA transfection was confirmed by real-time qPCR analysis of PANC-1 cells (Figure 4A). Furthermore, immunoblotting revealed that phosphorylation of STAT3 in sphere cells was inhibited in IL-6 or LIF knockdown PANC-1 sphere cells (Figure 4B), indicating that the gp130/STAT3 pathway is activated in PANC-1 sphere cells by the autocrine/paracrine action of IL-6 or LIF. Real-time qPCR analysis demonstrated that the expression levels of stemness markers other than *CD24* were reduced in IL-6 or LIF knockdown PANC-1 sphere cells compared to those in control sphere cells (Figure 4C). Invasion assays revealed that the number of invading cells was significantly reduced in IL-6 or LIF knockdown PANC-1 sphere cells compared to that in the control cells (Figure 4D). Real-time qPCR and western blot analyses demonstrated that MT1-MMP expression levels were significantly reduced in IL-6 or LIF knockdown PANC-1 sphere cells (Figure 4E,F). These results indicate that the autocrine/paracrine IL-6 or LIF/gp130/STAT3 pathways are involved in stemness and invasion of PANC-1 sphere cells.

### 3.5. Correlation between gp130/STAT3 and the TGF/Smad Pathway

**Figure 5:**
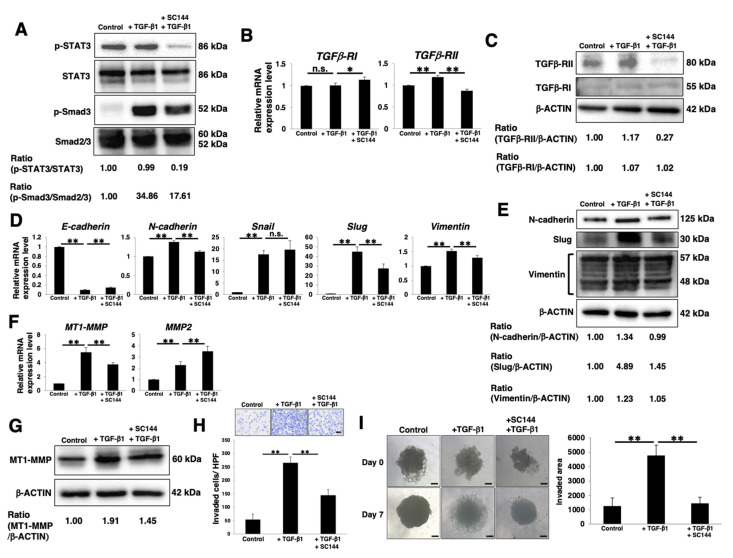
Correlation between gp130/STAT3 and the TGF/Smad pathway. PANC-1 cells were cultured for 4 days in 3D (sphere) conditions with or without 1 μM SC144 and further incubated for 3 days with or without 10 ng/mL TGF-β1. The cells were harvested and then used in following experiments. (**A**) Western blot analysis of p-gp130, gp130, p-STAT3, and STAT3. Relative band intensity is presented. (**B**) Real-time qPCR analysis of *TGFβ-RI* and *TGFβ-RII*. Results are normalized to values obtained for control cells (value = 1). Results are presented as means ± SD from three independent experiments. (**C**) Western blot analysis of TGFβ-RI, TGFβ-RII and β-ACTIN. Relative band intensity is presented. (**D**) Real-time qPCR analysis of EMT markers. Results are normalized to values obtained for control cells (value = 1). Results are presented as means ± SD from three independent experiments. (**E**) Western blot analysis of EMT markers and β-ACTIN (control). The relative band intensity is presented. (**F**) Real-time qPCR analysis of *MT1-MMP* and *MMP2*. Results are normalized to values obtained for control cells (value = 1). Results are presented as means ± SD from three independent experiments. (**G**) Western blot analysis of MT1-MMP and β-ACTIN. Relative band intensity is presented. (**H**) Matrigel invasion assays in PANC-1 sphere cells. Representative results from measurements of 12 fields are presented. Scale bar: 100 μm. (**I**) 3D invasion assay. Scale bar: 5 μm. The histograms indicate the invaded area. Results are presented the as means ± SD from four sphere images. * *p* < 0.05; ** *p* < 0.01, n.s.: not significant.

Several previous studies have demonstrated a two-way interplay between STAT3 and TGF-β signaling pathways [55,56,57]; however, many of these reports are controversial and the underlying mechanisms have not been fully elucidated. Furthermore, the correlation between the gp130/STAT3 and TGF/Smad pathways in the context of PDAC spheres (CSC-like cells) has not yet been clarified. Therefore, we examined whether the gp130/STAT3 pathway modulates activation of the TGF/Smad pathway to facilitate EMT induction in PDAC CSC-like cells. Western blot analysis revealed reduced phosphorylation of Smad3 in SC144-treated PANC-1 and PK-1 sphere cells, which exhibited reduced p-STAT3 levels compared to levels in sphere cells treated only with TGF-β1 (Figure 5A and Figure S3A, respectively), thus suggesting that the gp130/STAT3 pathway positively affects the TGF/Smad pathway. In SC144-treated PANC-1 and PK-1 sphere cells, TGFβ-RII mRNA and protein levels were reduced compared to those in sphere cells that were not treated with SC144 (Figure 5B,C and Figure S3B,C). These results suggest that the expression of TGFβ-RII via the gp130/STAT3 pathway may be required for the activation of the TGF/Smad pathway in PDAC CSC-like cells. Real-time qPCR analysis of EMT markers demonstrated that the reduction in *E-cadherin* and upregulation of *N-cadherin*, *Slug*, and *vimentin* in response to TGF-β1-treatment was attenuated in SC144-treated PANC-1 sphere cells (Figure 5D). Furthermore, western blot analysis for N-cadherin, Slug, and vimentin confirmed the changes observed at the mRNA level (Figure 5E). In contrast, the reduction in *E-cadherin* in response to TGF-β1-treatment was only attenuated in SC144-treated PK-1 sphere cells (Figure S3D). In SC144-treated PANC-1 sphere cells, upregulation of MT1-MMP in response to TGF-β1-treatment was attenuated at both the mRNA and protein levels (Figure 5F,G). In PK-1 sphere cells, there were no significant changes in the expression of MT1-MMP even after TGF-β1-treatment (Figure S3E). Next, we investigated one of the EMT features, specifically the invasion ability. Invasion assays revealed that the number of invading cells significantly increased in TGF-β1-treated PANC-1 sphere cells compared with that of control cells, and this increase was attenuated by SC144 treatment (Figure 5H). Furthermore, the 3D-invasion assay revealed that the increase in the invasion of TGF-β1-treated PANC-1 and PK-1 sphere cells was significantly inhibited by SC144-treatment (Figure 5I and Figure S3F). Taken together, these results indicate that the gp130/STAT3 pathway in CSC-like cells modulates the activation of the TGF/Smad pathway to facilitate EMT induction via regulation of TGFβ-RII expression, ultimately leading to increased invasion after the induction of EMT.

### 3.6. Autocrine/Paracrine IL-6 or LIF/gp130/STAT3 Pathways Regulate H19 Expression

**Figure 6:**
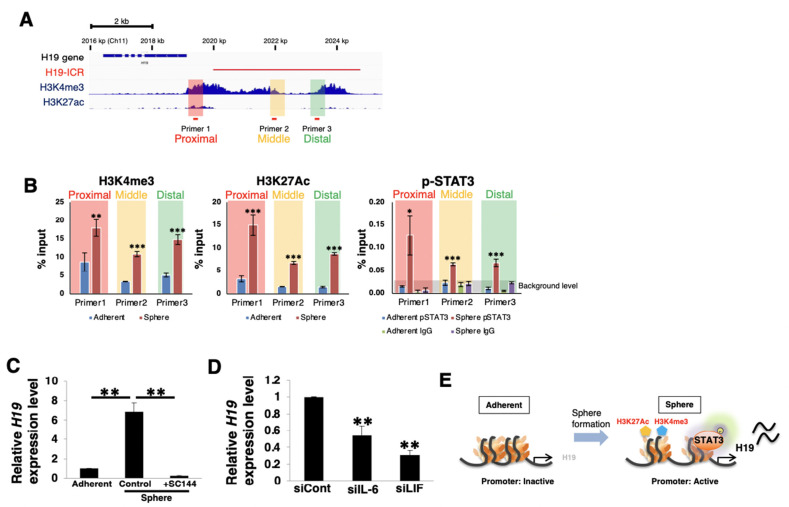
Autocrine/paracrine IL-6 or LIF/gp130/STAT3 pathways regulate *H19* expression. (**A**) Schematic representation of the proximal region of the human *H19* gene and RNA (human genome: hg19 ch12). Histogram images of H3K27me3 and H3K27Ac in PANC-1 cells were obtained from chromatin immunoprecipitation (ChIP)-atlas (http://chip-atlas.org/, accessed on 18 January 2022). The red line displays the ICR of *H19*. Three sets of PCR primers designated as Primer 1 (proximal region), Primer 2 (middle region), and Primer 3 (distal region) on the predicted *H19* promoter region were used for analysis of ChIP. (**B**) ChIP assays for PANC-1 after sphere formation were performed using anti-H3K27me3, H3K27Ac, and p-STAT3 antibodies and control IgG, and this was followed by real-time qPCR using the designed primer sets specific for the *H19* promoter. The data were normalized according to the percent input method. Data are presented as means ± SD from three independent experiments (* *p* < 0.05, ** *p* < 0.01, *** *p* < 0.005 vs. adherent cells). (**C**) Real-time qPCR analysis of *H19* in PANC-1 cells cultured for 7 days in 2D (adherent) or 3D (sphere) conditions with or without 1 μM SC144 for 7 days. Results are normalized to values obtained for adherent cells (value = 1). Results are presented as means ± SD from three independent experiments. ** *p* < 0.01 (**D**) Real-time qPCR analysis of *H19* in PANC-1 cells cultured for 7 days in 3D (sphere) conditions after siRNA transfection. Results are normalized to values obtained for siCont transfected sphere cells (value = 1). Results are presented as means ± SD from three independent experiments (**p < 0.01 vs. siCont transfected sphere cells). (**E**) Schema of epigenetic regulation on the predicted *H19* promoter region during sphere formation of PDAC cells. In the adherent state, the *H19* promoter region is closed and the *H19* expression level is low, while in the sphere formation state, this promoter is activated and p-STAT3 can access this region to facilitate the induction of *H19* expression.

*H19* is a genomic imprinting gene that is regulated by methylation in the imprinting control region (ICR) of the paternal allele and possesses a promoter/enhancer that induces transcription of the maternal allele. First, we estimated the promoter region near the *H19* gene in pancreatic cancer using ChIP-atlas (http://chip-atlas.org/, accessed on 18 January 2022) [58], and we identified the H3K4me3/H3K27Ac enrichment region that displays an active promoter region upstream of the transcriptional start site (TSS) of *H19* (Figure 6A). Next, we performed a ChIP assay to examine whether the gp130/STAT3 pathway affects *H19* expression under 2D (adherent) or 3D (sphere) culture conditions. Primer sets were designed at the predicted promoter regions (proximal, middle, and distal), as indicated by the red bars in the histogram in Figure 6A. The ChIP assay demonstrated that H3K4me3 and H3K27Ac enrichment in PANC-1 cells was increased in 3D-culture conditions compared to those in 2D-culture conditions (Figure 6B), thus indicating that sphere formation induced the active state of the *H19* promoter. Furthermore, we observed that p-STAT3 bound to the promoter only under 3D-culture conditions in PANC-1 and PK-1 cells (Figure 6B and Figure S4A). These results suggest that activated STAT3 in sphere cells could access the active promoter region of *H19* and contribute to its transcription. Real-time qPCR analysis demonstrated that *H19* expression was upregulated in PANC-1 and PK-1 sphere cells, and this expression was attenuated by SC144 treatment (Figure 6C and Figure S4B). Furthermore, IL-6 or LIF stimulation of PANC-1 cells in 2D culture conditions did not upregulate *H19* expression, whereas the reduction in IL-6 and LIF also attenuated *H19* expression in PANC-1 sphere cells (Figure 6D and Figure S5). Therefore, these results indicate that autocrine/paracrine IL-6 or LIF/gp130/STAT3 pathways contribute to *H19* expression in PDAC CSC-like cells (Figure 6E).

### 3.7. Contribution of H19 to EMT and Invasion in PDAC Sphere Cells

**Figure 7:**
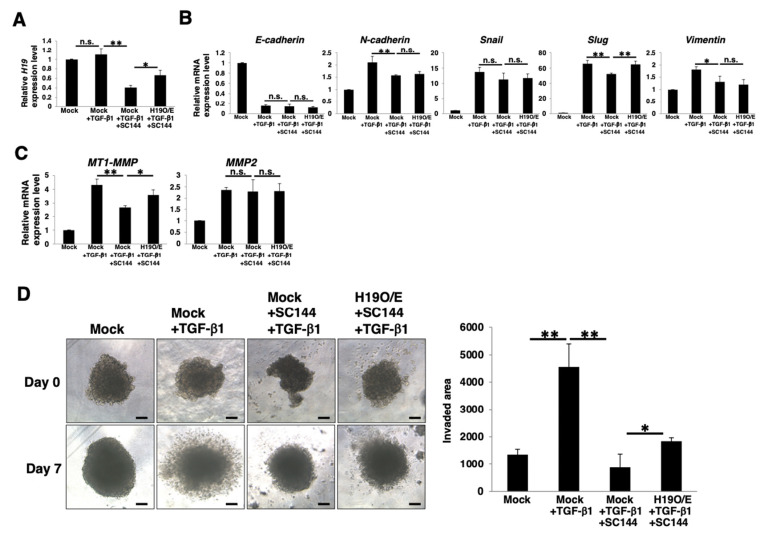
Contribution of *H19* to EMT and invasion in PDAC sphere cells. PANC-1 cells stably transfected with mock or *H19* expression vector were cultured for 4 days in 3D (sphere) conditions with or without 1 μM SC144 and then further incubated for 3 days with or without 10 ng/mL of TGF-β1. The cells were harvested and then used in the following experiments. (**A**) Real-time qPCR analysis of *H19*. Results are normalized to values obtained for mock-transfected sphere cells (value = 1). Results are presented as means ± SD from three independent experiments. (**B**) Real-time qPCR analysis of EMT markers. Results are normalized to values obtained for mock-transfected sphere cells (value = 1). Results are presented as means ± SD from three independent experiments. (**C**) Real-time qPCR analysis of *MT1-MMP* and *MMP2*. Results are normalized to values obtained for mock-transfected sphere cells (value = 1). Results are presented as means ± SD from three independent experiments. (**D**) 3D-invasion assay. Scale bar: 5 μm. The histograms indicate the invaded area. Results are presented as means ± SD from four sphere images. * *p* < 0.05; ** *p* < 0.01, n.s.: not significant, O/E: overexpression.

We examined whether *H19* expression via the gp130/STAT3 pathway contributes to the EMT and invasion of PDAC cells. In *H19*-overexpressed PANC-1 sphere cells, the expression level of *H19* was higher than that in mock-transfected sphere cells even after SC144-treatment (Figure 7A). Real-time qPCR analysis of EMT markers revealed that the reduction of *Slug* after TGF-β1-treatment was only rescued in *H19*-overexpressed PANC-1 sphere cells (Figure 7B). The reduction of *MT1-MMP* was also rescued in *H19*-overexpressed sphere cells (Figure 7C). The 3D-invasion assay demonstrated that the reduction in invasion in SC144-treated mock-transfected PANC-1 sphere cells was partially rescued in *H19*-overexpressed sphere cells (Figure 7D). These results indicate that *H19*, regulated by the gp130/STAT3 pathway, partially contributes to EMT induction and invasion through MT1-MMP expression in PDAC CSC-like cells.

### 3.8. The Correlation of gp130/STAT3 Pathway-Related Factor Expression with Overall Survival and H19 Expression Correlated with gp130/STAT3 Pathway-Related Factors in PDAC Patients

**Figure 8:**
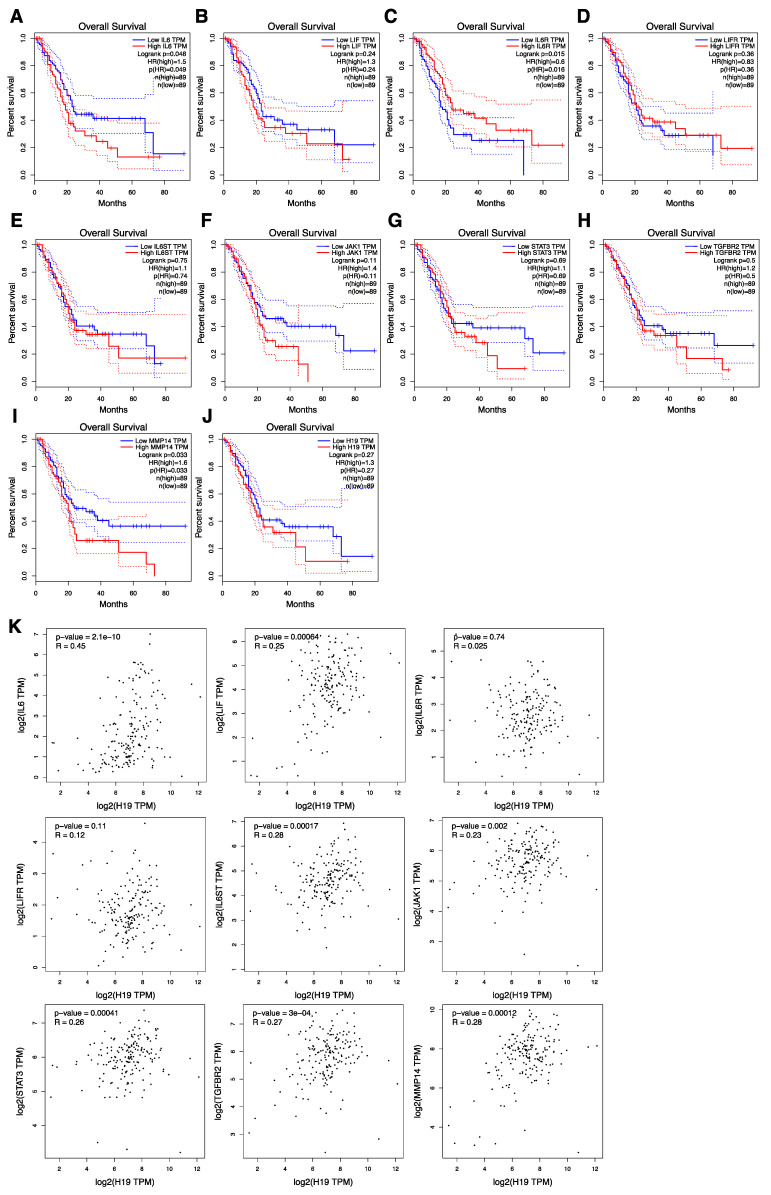
The correlation of gp130/STAT3 pathway-related factor expression with overall survival and *H19* expression correlated with gp130/STAT3 pathway-related factors in patients with PDAC. Each survival curve was presented according to the online database GEPIA. (**A**) *IL-6*, (**B**) *LIF*, (**C**) *IL-6R*, (**D**) *LIFR*, (**E**) *gp130 (IL-6ST)*, (**F**) *JAK1*, (**G**) *STAT3*, (**H**) *TGFβ-RII (TGFBR2)*, (**I**) *MT1-MMP (MMP14)*, and (**J**) *H19*. (**K**) The correlation of *H19* and gp130/STAT3 pathway-related factor expression in patients with PDAC was assessed using Spearman rank correlation analysis according to the online database GEPIA.

Finally, we investigated whether gp130/STAT3 pathway-related factors correlated with prognosis in patients with PDAC. As presented in Figure 8A,F–J, higher expression levels of *IL-6* and *MT1-MMP* were significant, and *JAK1*, *STAT3*, *TGFβ-RII*, and *H19* were correlated with poorer overall survival in patients with PDAC. Next, to clarify the relationship between *H19* and the gp130/STAT3 pathway in patients with PDAC, we focused on the correlations between *H19* expression and gp130/STAT3 pathway-related factors. Data from GEPIA revealed significant correlations between *H19* and gp130/STAT3 pathway-related factors, such as *IL-6*, *LIF*, *gp130*, *JAK1*, *STAT3*, *TGFβ-RII*, and *MT1-MMP* (Figure 8K). Therefore, these results suggest that autocrine/paracrine IL-6 or the LIF/gp130/STAT3 pathway, including *H19* expression, is correlated with poor prognosis in PDAC.

## 4. Discussion

The involvement of IL-6 or the LIF/gp130/STAT3 pathway in PDAC pathogenesis has been demonstrated previously [59]. In PDAC 2D-culture cells, knockdown of IL-6 resulted in apoptosis, growth inhibition, and reduced invasion [60]. In in vivo experiments, the growth of human PDAC cells was significantly reduced by pharmacological inhibition of IL-6 signaling [61]. Inhibition of LIF signaling by genetic LIFR deletion, LIF knockdown, or treatment with LIF neutralizing monoclonal antibodies leads to a reduction in tumor progression in mouse PDAC models and decreases tumor engraftment and growth in PDAC xenograft models [62,63]. In prostate cancer, glioma, endometrial cancer, and breast cancer, the IL-6/gp130/STAT3 signaling pathway has been demonstrated to be involved in the regulation of CSC properties such as self-renewal, stemness marker expression, and tumor cell growth [22,23,24,25]. In the LIF/gp130/STAT3 pathway, the relationship with CSCs has not been directly demonstrated; however, recent evidence indicates that LIF functions as a growth factor to promote the growth and invasion of osteosarcoma [64] and that LIF/gp130/STAT3 signaling via autocrine and paracrine mechanisms is upregulated in tumors [65,66,67]. In this study (Figure 1, Figure 2, Figure 3 and Figure 4, Figure S1), we observed that the autocrine/paracrine IL-6 or the LIF/gp130/STAT3 pathway is active in PDAC CSCs that are formed under 3D-culture conditions and that this pathway contributes to CSC growth, stemness marker expression, and invasion ability in two of four PDAC cell lines (PANC-1 and PK-1). In contrast, there were no obvious effects on anticancer drug resistance after inhibition of the gp130/STAT3 pathway by SC144 treatment. It is suggested that no reduction in transporters other than ABCC2 may affect anti-cancer drug excretion in SC144-treated cells by inhibiting the gp130/STAT3 pathway. As STAT3 directly controls the expression of MT1-MMP [68], autocrine/paracrine IL-6, or the LIF/gp130/STAT3 pathway, regulates MT1-MMP expression, ultimately leading to the invasiveness of PDAC CSCs. The OSM/gp130/STAT3 pathway is also correlated with cancer malignancy. The binding of OSM to OSM receptor (OSMR) causes OSMR-gp130 dimerization, which is required to produce oncogenic signaling cues for prolonged STAT3 activation. Anti-OSMR antibody inhibited the growth of ovarian cancer cells in vitro and in vivo by suppressing STAT3 activation [69]. OSM-mediated signaling has also been shown to contribute to the growth and invasion of triple-negative breast cancer [70]. Moreover, OSM and OSMR are overexpressed in PDAC compared with their expression in the normal pancreas [71]. OSM-treated PDAC cells exhibit CSC properties, enhanced tumorigenicity, and increased metastasis through STAT3 activation [71]. Here, we demonstrate the autocrine/paracrine contribution of IL-6 or LIF to STAT3 activation, leading to the maintenance of CSC properties; however, we cannot exclude the possible involvement of the autocrine/paracrine OSM/gp130/STAT3 pathway in PDAC CSCs; therefore, further investigation is required.

Several previous studies have demonstrated a relationship between STAT3 and TGF-β signaling pathways [55,56,57]; however, many of these studies have yielded controversial results. Activated STAT3 was found to sequester Smad3 from the Smad nucleoprotein complex, thus suppressing TGF-β signaling [52]. In our study (Figure 5 and Figure S3), TGF-β1/Smad signaling, which resulted in EMT, was induced even in STAT3-activated PDAC CSCs. In addition to the activation of STAT3 through IL-6 or the LIF/gp130 pathway, STAT3 can be activated by a wide range of ligands that bind to cytokines, growth factors, or G-protein-coupled receptors [72]. In SC144-treated PDAC CSCs, STAT3 activation was fully inhibited, suggesting that the gp130/STAT3 pathway primarily contributes to the activation of STAT3 in PDAC CSCs. If other pathways for STAT3 activation are functionally dependent on cellular context, TGF-β signaling may be inhibited by activated STAT3. We speculate that the balance between p-Smad3 levels and sequestration of Smad3 from the Smad nucleoprotein complex due to STAT3 activation may lead to TGF-β signaling-mediated EMT induction or growth promotion via the suppression of TGF-β signaling.

We recently reported that PDAC cell lines are heterogeneous and that 3D cultures enhance differences in cell morphology and function [51]. PDAC cell lines include epithelial-like and quasi-mesenchymal cells [73]. The PANC-1 cells used in this study were classified into a mesenchymal phenotype expressing low E-cadherin and high vimentin, while PK-1, PK-59, and PK-8 cells are epithelial cells possessing the opposite expression patterns [74]. As presented in Figure 5D and Figure S3D, there were differences in EMT induction between PANC-1 and PK-1 cells. It is possible that these genetic and phenotypic differences affected the differences between the two cell lines. Further research focusing on the differences between PDAC cell lines is required.

It has been demonstrated that miR-301a suppresses the expression of the suppressor of cytokine signaling 5 (SOCS5), thus leading to JAK/STAT3 activation and resulting in increased invasion and metastasis of PDAC cells [75]. Furthermore, *H19*-derived miR-675-3p promotes JAK/STAT3 activation by directly targeting SOCS5 [76]. As demonstrated in this study, elevated *H19* expression in PDAC CSCs may promote positive feedback constitutive activation of the gp130/STAT3 pathway by reducing the expression of SOCS5. In contrast, inhibition of gp130 may lead to a negative loop in the attenuated gp130/STAT3 pathway by promoting SOCS5 expression through reduced expression of *H19*-derived miR-675-3p. In CRC, *H19* functions as an miR-22-3p sponge to increase the expression of MT1-MMP, thus promoting lung metastasis [77]. Therefore, it is possible that the upregulation of MT1-MMP in PDAC CSCs (Figure 3) may be dependent on the reduction in miR-22-3p availability by *H19*. In this study, we revealed that TGFβ-RII expression was regulated by the gp130/STAT3 pathway (Figure 5). A previous study demonstrated that *H19* contributes to TGFβ-RII transcription by regulating transcription factor expression through miRNA sponges [78]. Further studies are required; however, it is possible that *H19* expressed through the gp130/STAT3 pathway functions as an miRNA sponge to facilitate the upregulation of TGFβ-RII expression in PDAC CSCs. The CSC characteristics of various PDAC cell subpopulations correlate with their aggressive and metastatic phenotypes. CD133+/CXCR4+ PDAC CSCs [79] are tumorigenic. CD133 enhances N-cadherin expression via the Src/Slug signaling axis to facilitate EMT in PDAC cells [80]. Moreover, CD133 initiates the expression and transcriptional activity of HIF-1α, which promotes PDAC cell migration and metastasis by inducing EMT gene transcription or autophagy under hypoxic conditions [81,82]. Furthermore, hypoxia induces a positive feedback loop of constitutive activation between nestin expression and the TGF-β1/Smad4 pathway [83]. Nestin, a pancreatic CSC marker, is necessary to drive EMT and promote PDAC cell invasion [84,85]. Therefore, we speculated that *H19* expressed in PDAC CSCs via the gp130/STAT3 pathway contributes to the aggressiveness of PDAC through TGF-β1/Smad-mediated EMT.

As presented in Figure 8, high expression of gp130/STAT3 pathway-related factors was associated with survival rate, and *H19* expression was correlated with the expression of these factors in PDAC patients. Therefore, the gp130/STAT3 pathway, including *H19*, may be a promising target for the treatment of PDAC, including CSCs. From our in vitro experiments, p-gp130 is considered a new CSC marker in PDAC. We then performed immunohistochemical staining for p-gp130 in human PDAC tissues; however, no significant results were obtained (data not shown). We believe that it may be difficult to detect p-gp130 due to the small population of p-gp130-positive CSCs, and there are few anti-p-gp130 antibodies suitable for tissue staining. In the future, it will be necessary to verify whether inhibition of the gp130/STAT3 pathway is effective for the attenuation of metastasis or the prevention of recurrence in pancreatic cancer through in vivo experiments targeting gp130. We established stable gp130 knockdown PDAC cells, in which reduced expression of gp130 was confirmed by FACS analysis; however, these cells exhibited promotion of invasiveness, contrary to our results (Figure S6). The membrane protein gp130 forms a receptor complex with not only IL-6R and LIFR but also with IL-11 receptor [86], OSMR, ciliary neurotrophic factor receptor [87], and WSX-1 [88]. The constitutive reduction of gp130 is suggested to affect the formation of these complexes and may lead to changes in STAT3 activation, as well as other STAT pathways. Alternatively, gp130 is expressed in lipid rafts, in which signal receptors and integrins are present; therefore, there is a possibility that reduced expression of gp130 may affect the expression of these lipid raft-associated proteins, leading to the observed increased invasion ability. This aspect needs to be examined in the future. Therefore, we believe that gp130 inhibitors such as SC144 are appropriate for further in vivo experiments. In vivo mouse experiments using SC144 have demonstrated that oral administration of SC144 delays tumor growth in a mouse xenograft model of human ovarian cancer, without significant toxicity to normal tissues [89]. Bazedoxifene, approved by the Food and Drug Administration (FDA) as a selective estrogen modulator for osteoporosis treatment [90], has been demonstrated to be a new drug that inhibits the IL-6/gp130/STAT3 pathway [91]. In addition to osteoporosis treatment, in vivo mouse experiments in breast cancer, blastoma, pancreatic cancer, and cervical cancer have been conducted, and inhibition of tumor formation has been reported [92,93,94,95,96]. Furthermore, preliminary tests in patients with pancreatic or gastric cancer treated with bazedoxifene revealed tumor marker reduction, metabolic regression, tumor size reduction, and improved quality of life [97].

## 5. Conclusions

**Figure 9:**
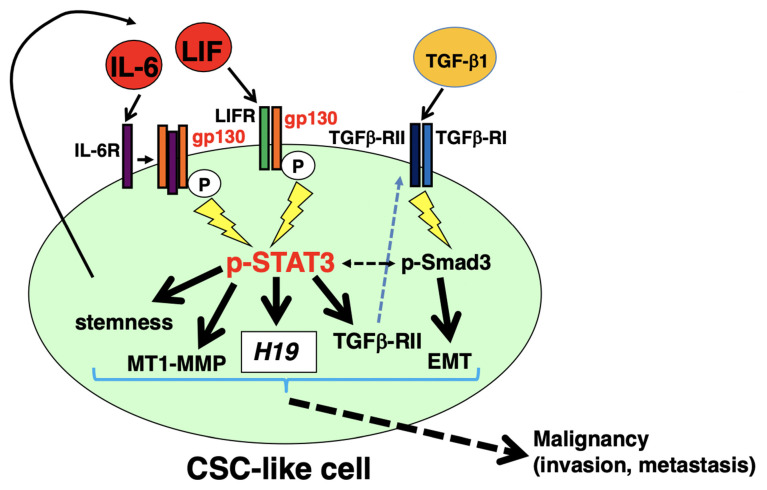
Schematic representation of autocrine/paracrine IL-6 or the LIF/gp130/STAT3 pathway in PDAC sphere cells. In PDAC sphere cells in which CSCs are enriched (CSC-like cells), binding of autocrine/paracrine IL-6 or LIF to each receptor (IL-6R or LIFR, respectively) induces gp130 homodimerization or LIFR/gp130 complex formation to thereby activating JAKs followed by phosphorylation of gp130, ultimately leading to STAT3 activation. This pathway contributes to the maintenance of stemness features and expression of MT1-MMP and TGFβ-RII. Expression of TGFβ-RII via the gp130/STAT3 pathway affects TGF-β1/Smad signaling to promote EMT induction. Additionally, p-STAT3 can access the active promoter region of *H19* and contribute to its transcription. Therefore, autocrine/paracrine IL-6 or the LIF/gp130/STAT3 pathway in PDAC CSC-like cells is believed to eventually lead to invasion and metastasis, both of which are hallmarks of malignancy.

In this study, we demonstrated that the autocrine/paracrine IL-6 or LIF/gp130/STAT3 pathways contribute to the maintenance of stemness features and MT1-MMP expression, and contribute to TGF-β1/Smad signaling-mediated EMT and invasion by regulating TGFβ-RII expression in PDAC CSC-like cells (Figure 9). Furthermore, we demonstrated that p-STAT3 could access the active promoter region of *H19* and contribute to its transcription (Figure 9). Consequently, the autocrine/paracrine IL-6 or LIF/gp130/STAT3 pathways in PDAC CSC-like cells, including *H19* expression, are considered to eventually lead to invasion and metastasis, both of which are hallmarks of malignancy. Therefore, the inhibition of the gp130/STAT3 pathway represents a promising strategy for PDAC therapy.

## Data Availability

The datasets used and/or analyzed during the current study are available from the corresponding author upon reasonable request.

## Supplementary Materials

The following are available online at https://www.mdpi.com/article/10.3390/cancers14092055/s1, Table S1: Primers list, Table S2: List of primary antibodies used for immunoblotting, Table S3: Primers list for ChIP assay, Figure S1: Active gp130/STAT3 pathway in other PDAC sphere cells., Figure S2: Active gp130/STAT3 pathway involved in stemness and invasion of PK-1sphere cells., Figure S3: Correlation between gp130/STAT3 and TGF/Smad pathway in PK-1 cells., Figure S4: Autocrine/paracrine IL-6 or LIF/gp130/STAT3 pathway regulate *H19* expression in PK-1 sphere cells., Figure S5: *H19* expression in IL-6- or LIF-stimulated PANC-1 cells; Figure S6: Stable knockdown of gp130 in PANC-1 cells; Figure S7: Western blots.
