# Supplementary material for: Gp130-Mediated STAT3 Activation Contributes to the Aggressiveness of Pancreatic Cancer through H19 Long Non-Coding RNA Expression
Source: Cancers (Basel). 2022 Apr 19;14(9):2055. doi: 10.3390/cancers14092055 (PMC9100112; doi:10.3390/cancers14092055)
Supplement: Supplementary file 1 [file cancers-14-02055-s001.zip › cancers-1689959-supplementary.pdf]

Table S1. Primers list.

Gene.	Forward primer	Reverse primer
Oct3/4	GGAGGAAGCTGACAACAATGAAA	GGCCTGCACGAGGGTTT
Nanog	CCAAAGGCAAACAACCCACTT	CGGGACCTTGTCTTCCTTTTT
ALDH1	GAGCCCTTGCATTGTGTTAGC	CCATGGTGTGCAAATTCAACAG
Nestin	TCCTGCTGTAGATGCAGAGATCAG	ACCCTGTGTCTGGAGCAGAGA
CD24	TCCAACTAATGCCACCACCAA	GACCACGAAGAGACTGGCTGTT
CD44v9	AGCAGAGTAATTCTCAGAGCTT	TGCTTGATGTCAGAGTAGAAGT
β-ACTIN	GGTCATCACCATTGGCAATGAG	TACAGGTCTTTGCGGATGTCC
ABCG2	TGGCTGTCATGGCTTCAGTACT	CATTATGCTGCAAAGCCGTAAA
ABCB1	TGACAGCTACAGCACGGAAG	TCTTCACCTCCAGGCTCAGT
ABCC1	GAGAGTTCCAAGGTGGATGC	AGGGCCCAAAGGTCTTGTAT
ABCC2	TACCAATCCAAGCCTCTACC	AGAATAGGGACAGGAACCAG
MT1-MMP	GAAGGATGGCAAATTCGTCTTC	AGGGACGCCTCATCAAACAC
MMP2	GCGGCGGTCACAGCTACTT	TTCAGACTTTGGTTCTCCAGCTT
IL-6	AAAAAGGCAAAGAATCTAGATGCAA	GTCAGCAGGCTGGCATTTGT
LIF	TCTTGGCGGCAGGAGTTG	CCGCCCCATGTTTCCA
IL-6R	TGAGCTCAGATATCGGGCTGAAC	CGTCGTGGATGACACAGTGATG
LIFR	TGGAACGACAGGGGTTCAGT	GAGTTGTGTTGTGGGTCACTAA
gp130	AGGACCAAAGATGCCTCAAC	GAATGAAGATCGGGTGGATG
TGFβ-RI	GCAGAGCTGTGAAGCCTTGAGA	TGCCTTCCTGTTGACTGAGTTG
TGFβ-RII	ATGACATCTCGCTGTAATGC	GGATGCCCTGGTGGTTGA
E-cadherin	CCAGTGAACAACGATGGCATT	TGCTGCTTGGCCTCAAAAT
N-cadherin	TGGGAATCCGACGAATGG	GCAGATCGGACCGGATACTG
Snail	CCCCAATCGGAAGCCTAACT	GCTGGAAGGTAAACTCTGGATTAGA
Slug	TGCGGCAAGGCGTTTT	TCTCCCCCGTGTGAGTTCTAA
Vimentin	TCCAAACTTTTCCTCCCTGAAC	GGGTATCAACCAGAGGGAGTGA

Table S2. List of primary antibodies for immunoblotting.

Primary antibody	Antibody dilution
Monoclonal rabbit anti-p-Smad3 (ab52903; Abcam, Cambridge, UK)	1:1000
Monoclonal rabbit anti-Smad2/3 (#8685; Cell Signaling Technology, Danvers, MA, USA)	1:1000
Monoclonal mouse anti-STAT3 (#9139; Cell Signaling Technology)	1:1000
polyclonal rabbit anti-p-STAT3 (Tyr705) (#9131; Cell Signaling Technology)	1:1000
polyclonal rabbit anti-gp130 (ab202850; Abcam)	1:1000
Monoclonal mouse anti-p-gp130 (sc-377572; Santa Cruz Biotechnology, Dallas, TX, USA)	1:1000
Polyclonal rabbit anti-transforming growth factor β receptor I (TGFβR-I) (SAB4502958; Sigma-Aldrich, St. Louis, MO, USA)	1:1000
Monoclonal rabbit anti-TGFβR-II (ab184948; Abcam)	1:1000
Monoclonal rabbit anti-membrane-type 1 matrix metalloproteinase (MT1-MMP) (ab51074; Abcam)	1:1000
Monoclonal mouse anti-N-cadherin (350802; BioLegend)	1:1000
Polyclonal rabbit anti-Slug (12129-1-AP; Proteintech Group, Inc, Rosemont, IL, USA)	1:1000
Monoclonal mouse anti-Vimentin (sc-373717; Santa Cruz Biotechnology)	1:1000
Monoclonal mouse anti-β-ACTIN (A5316; Sigma-Aldrich).	1:10000

Table S3. Primers list for ChIP assay.

		H19 ChIP-primer 1	
**Forward**	5'	GACCTCACGTTCCTGGAGAG	3
Reverse	5'	GCGGTCTTCAGACAGGAAAG	3
		H19 ChIP-primer 2	
Forward	5'	ATGTGGCTCCCATGAATGTC	3
Reverse	5'	GGCTCTTGCATAGCACATGA	3
		H19 ChIP-primer 3	
Forward	5'	GATATGGCCCGATACGAAGA	3
Reverse	5'	TTCCCCTTCTGTCTCACCAC	3

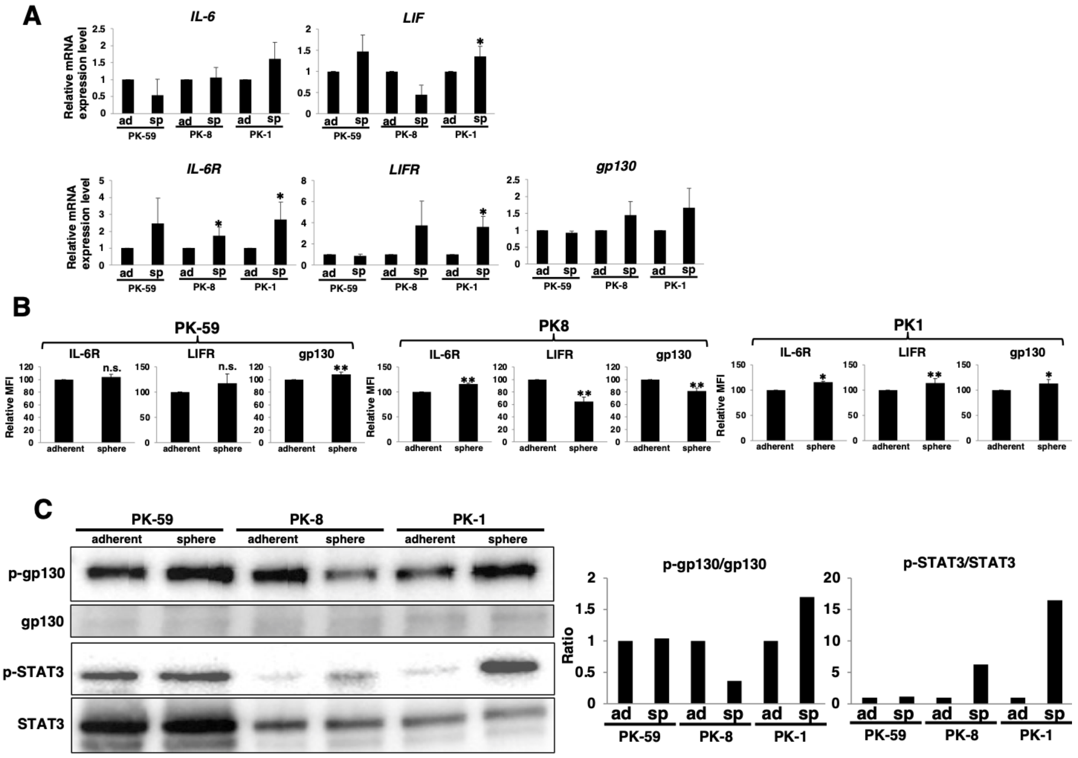

Figure S1. Active gp130/STAT3 pathway in other PDAC sphere cells. (**A**) Real-time qPCR analysis of *IL-6*, *LIF*, *IL-6R*, *LIFR*, and *gp130* in PDAC cells cultured in 2D (adherent) or 3D (sphere) conditions. Results are normalized to values obtained for adherent cells (value = 1). Results are presented as means ± SD from three independent experiments. (**B**) Cell surface levels of IL-6R, LIFR, and gp130 expression according to FACS analysis in PDAC cells cultured in 2D (adherent) or 3D (sphere) conditions. Mean fluorescence intensities (MFIs) relative to those of adherent cells are presented. Results are presented as means ± SD from three independent experiments. (**C**) Western blot analysis of p-gp130, gp130, p-STAT3, and STAT3 was performed in PDAC cells cultured in 2D (adherent) or 3D (sphere) conditions. The histograms indicate relative band intensity. * $p < 0.05$, ** $p < 0.01$, n.s.: not significant vs. adherent cells. ad: adherent, sp: sphere.

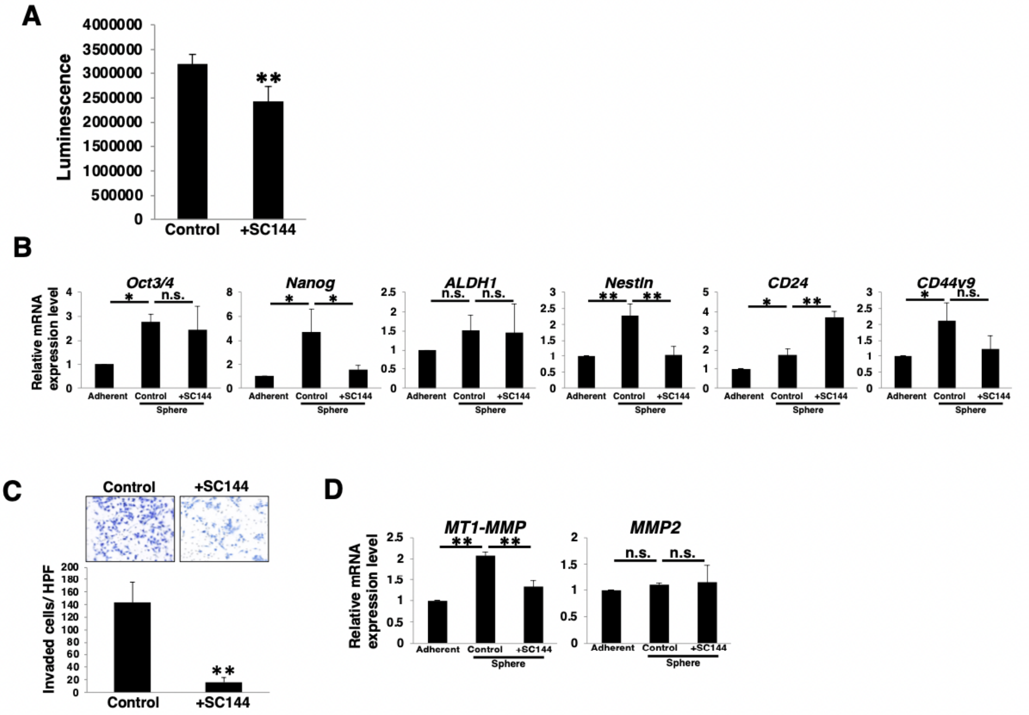

Figure S2. Active gp130/STAT3 pathway involved in stemness and invasion of PK-1 sphere cells. PK-1 cells were cultured for 7 days in 2D (adherent) or 3D (sphere) conditions with or without 1 μM SC144 for 7 days. The cells were harvested and then used in the following experiments. (**A**) ATP assay of PK-1 sphere cells. (**B**) Real-time qPCR analysis of stemness markers. Results are normalized to values obtained for adherent cells (value = 1). Results are presented as means ± SD from three independent experiments. (**C**) Matrigel invasion assays performed in PK-1 sphere cells. Representative results from measurements of 12 fields are presented. (**D**) Real-time qPCR analysis of *MT1-MMP* and *MMP2*. Results are normalized to values obtained for adherent cells (value = 1). Results are presented as means ± SD from three independent experiments. *$P < 0.05$; **$P < 0.01$, n.s.: not significant.

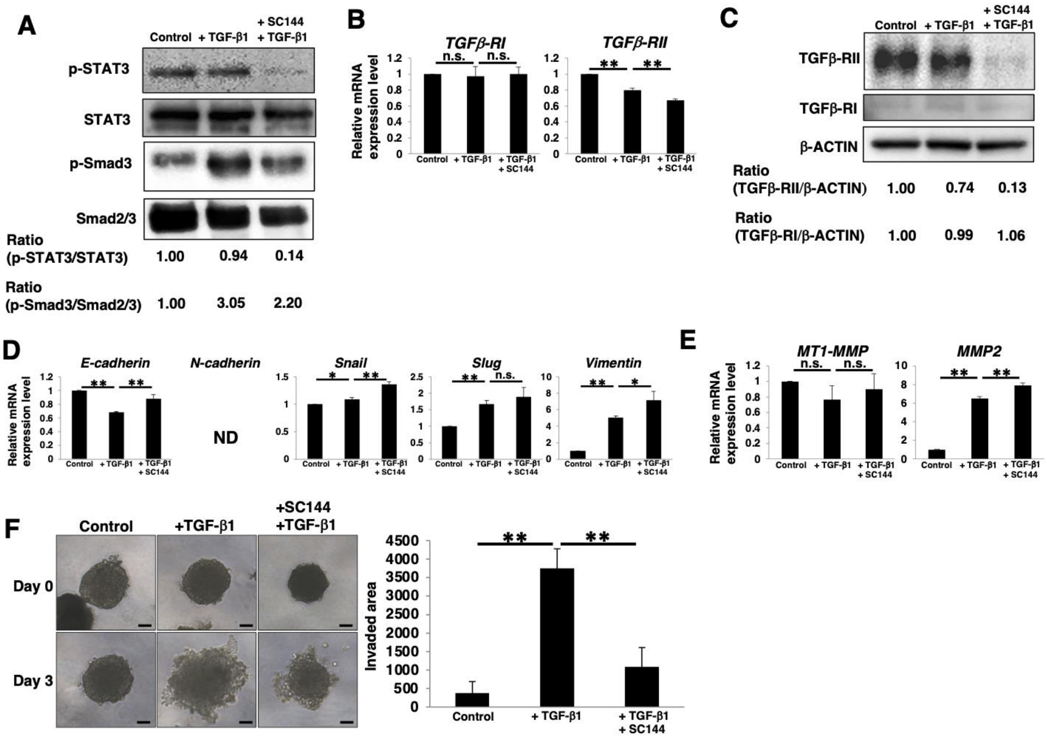

Figure S3. Correlation between gp130/STAT3 and the TGF/Smad pathway in PK-1 cells. PK-1 cells were cultured for 4 days in 3D (sphere) conditions with or without 1 μM SC144 and then further incubated for 3 days with or without 10 ng/ml TGF-β1. The cells were harvested and then used in the following experiments. (**A**) Western blot analysis of p-gp130, gp130, p-STAT3, and STAT3. Relative band intensity is presented. (**B**) Real-time qPCR analysis of *TGFβ-RI* and *TGFβ-RII*. Results are normalized to values obtained for control cells (value = 1). Results are presented as means ± SD from three independent experiments. (**C**) Western blot analysis of TGFβ-RI, TGFβ-RII, and β-ACTIN. Relative band intensity is presented. (**D**) Real-time qPCR analysis of EMT markers. Results are normalized to values obtained for control cells (value = 1). Results are presented as means ± SD from three independent experiments. (**E**) Real-time qPCR analysis of *MT1-MMP* and *MMP2*. Results are normalized to values obtained for control cells (value = 1). Results are presented as means ± SD from three independent experiments. (**F**) 3D invasion assay. The histograms indicate the invaded area. Results are presented as means ± SD from three sphere images. Scale bar: 2μm. *$P < 0.05$; **$P < 0.01$, n.s.: not significant.

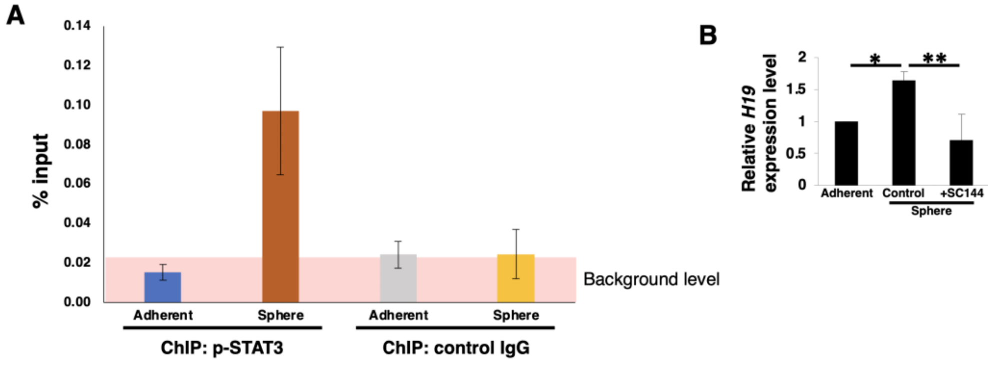

Figure S4. Autocrine/paracrine IL-6 or LIF/gp130/STAT3 pathways regulate *H19* expression in PK-1 sphere cells. (**A**) ChIP assays for PK-1 cells after sphere formation were performed using anti-p-STAT3 antibody and control IgG, and this was followed by real-time qPCR with the primer 1 for the *H19* promoter. The data were normalized according to the percent input method. Data are presented as means ± SD from two independent experiments. (**B**) Real-time qPCR analysis of *H19* in PK-1 cells cultured for 7 days in 2D (adherent) or 3D (sphere) conditions with or without 1 μM SC144 for 7 days. Results are normalized to values obtained for adherent cells (value = 1). Results are presented as means ± SD from three independent experiments. **P* < 0.05; ***P* < 0.01.

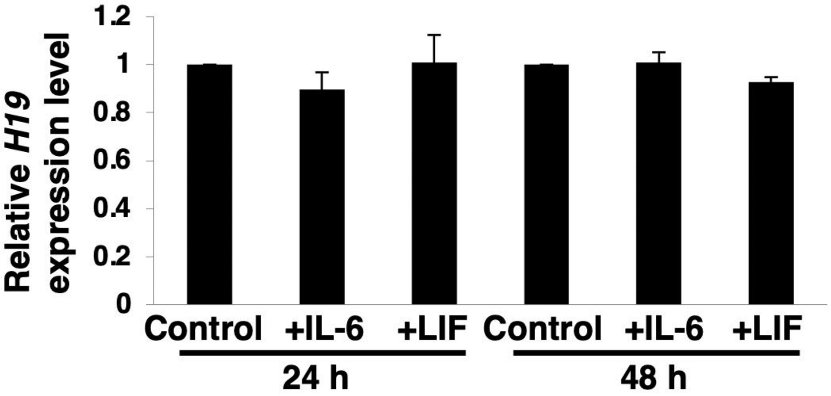

Figure S5. *H19* expression in IL-6- or LIF-stimulated PANC-1 cells. Real-time qPCR analysis of *H19* in PANC-1 cells cultured for 24 h or 48 h in a 2D (adherent) condition with or without 50 ng/ml IL-6 or 50 ng/ml LIF. Results are normalized to values obtained for non-treated cells (value = 1). Results are presented as means ± SD from triplicate measurements.

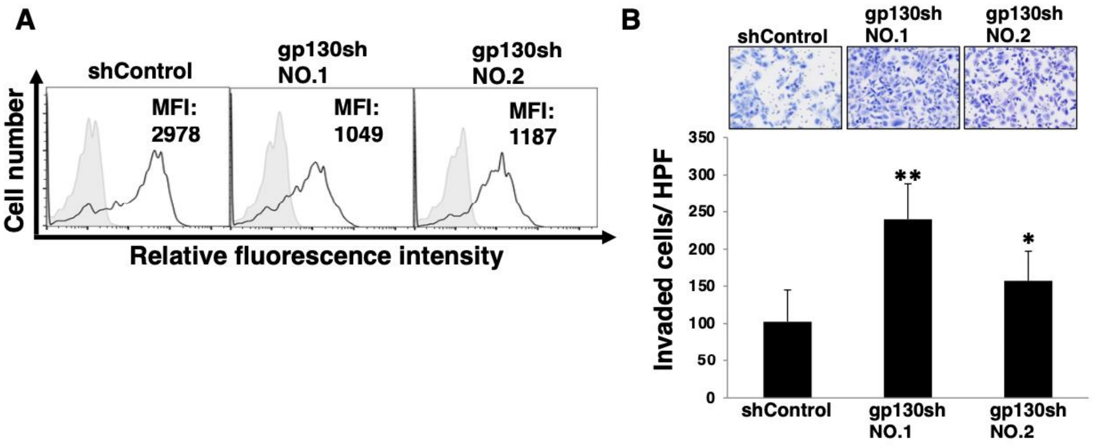

Figure S6. Stable knockdown of *gp130* in PANC-1 cells. Stable *gp130* shRNA-expressing PANC-1 cells were generated after transfection of *gp130* shRNA and scrambled shRNA vectors (pLKO.1-puro, Sigma-Aldrich Corporation) followed by selection on puromycin for approximately 1 month. (**A**) FACS analysis of gp130 expression in PANC-1 cells cultured in a 3D (sphere) condition. Controls are indicated by thin gray lines; the mean fluorescence intensity (MFI) is given for each graph. (**B**) Matrigel invasion assays performed in PANC-1 sphere cells. Representative results from measurements of 12 fields are presented. (*$p < 0.05$, **$p < 0.01$ vs. shControl-transfected sphere cells).

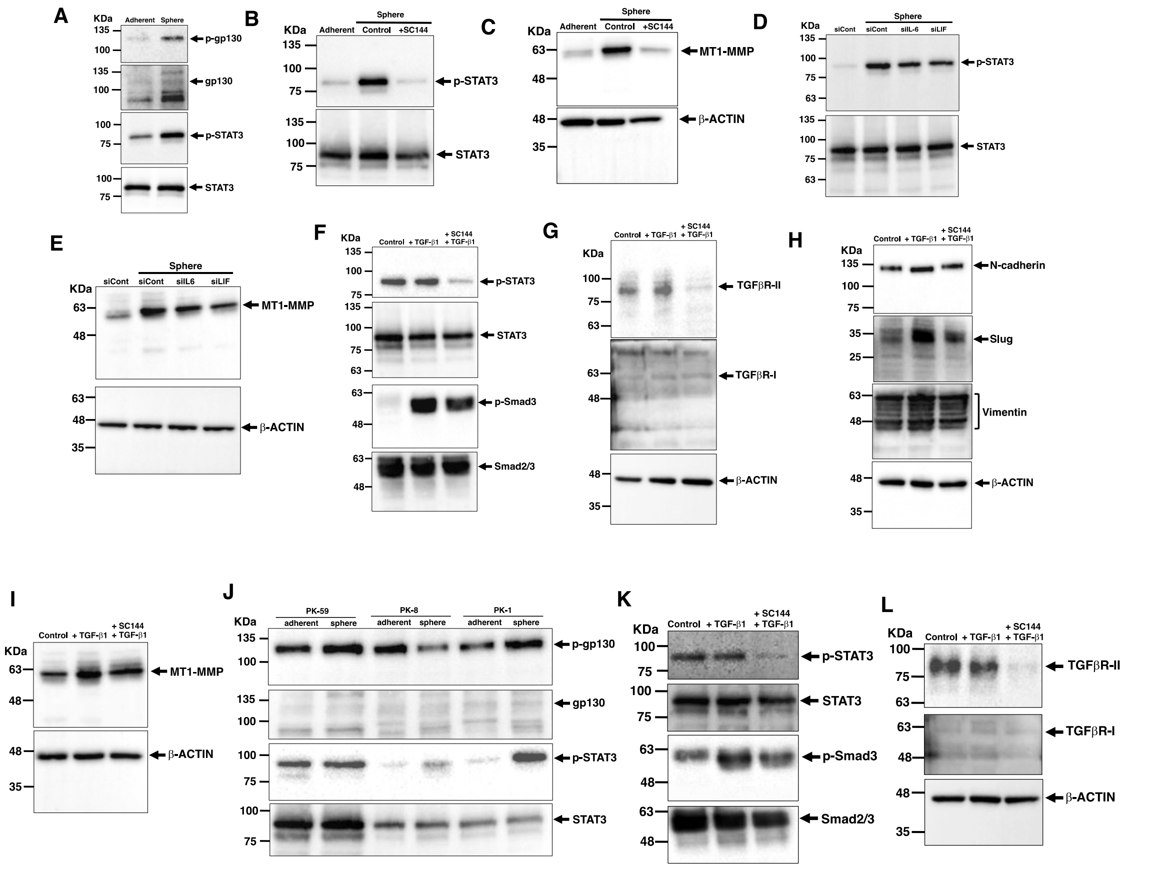

Figure S7. Western blots. Protein bands and molecular weight markers are presented for (**A**) Figure 1D, (**B**) Figure 3A, (**C**) Figure 3I, (**D**) Figure 4B, (E) Figure 4F, (**F**) Figure 5A, (**G**) Figure 5C, (**H**) Figure 5E, (**I**) Figure 5F, (**J**) Figure S1C, (**K**) Figure S3A, and (**L**) Figure S3C.
